# Association of pharmacokinetic biomarkers with early immune recovery following HLA-haploidentical hematopoietic cell transplantation

**DOI:** 10.3389/fimmu.2025.1694754

**Published:** 2025-12-05

**Authors:** Guofang Shen, Timothy W. Synold, Shanzay Khan, David A. Horne, Christopher G. Kanakry, Ryotaro Nakamura

**Affiliations:** 1 Department of Hematology and Hematopoietic Cell Transplantation, City of Hope, Duarte, CA, United States; 2 Department of Cancer Biology and Molecular Medicine, City of Hope, Duarte, CA, United States; 3 Center for Immuno-Oncology, Center for Cancer Research, National Cancer Institute, National Institutes of Health, Bethesda, MD, United States; 4 Department of Hematologic Malignancies Translational Science, City of Hope, Duarte, CA, United States

**Keywords:** post-transplant cyclophosphamide (PTCy), mycophenolate mofetil (MMF), regulatory T cells (Tregs), immune monitoring, pharmacokinetics, hematopoietic cell transplantation (HCT)

## Abstract

**Background:**

Prophylactic immunosuppressants for graft-versus-host disease (GVHD) in allogeneic hematopoietic cell transplantation (alloHCT), including post-transplant cyclophosphamide (PTCy) and mycophenolate mofetil (MMF), exhibit complex pharmacokinetic profiles. Interindividual variations in pharmacokinetic exposure to these immunosuppressants or their metabolites may interfere with treatment outcomes.

**Method:**

A feasibility study (n = 11) was conducted to investigate the pharmacokinetic/pharmacodynamic relationship in patients undergoing HLA-haploidentical alloHCT with standard high-dose PTCy (50 mg·kg^−1^·day^−1^ on days +3/+4) combined with MMF and tacrolimus or sirolimus. Blood samples were collected to assess the variability in pharmacokinetic biomarkers, including exposures [areas under the curve (AUCs)] to cyclophosphamide (Cy), carboxycyclophosphamide (cepm), *N*-dechloroethyl cyclophosphamide (dccy), 4-ketocyclophosphamide (ketocy), mycophenolic acid (MPA), and mycophenolic acid glucuronide (MPAG). Serial dynamic changes in immune cell populations, including regulatory T cells (Tregs), over the first 3 post-transplant weeks were monitored.

**Results:**

A transient reduction in the proliferation (Ki-67^+^) of activated (HLA-DR^+^) T cells coincided with Cy treatment. The ratio of Tregs to the CD4^+^ T-cell population increased in a time-dependent manner within the first 21 days post-transplant. We observed moderate interindividual variability across all pharmacokinetic biomarkers. Serum creatinine and blood urea nitrogen levels positively correlated with exposure to Cy and MMF metabolites, including cepm, MPA, and MPAG. Using correlation analysis, we further confirmed the negative association between pharmacokinetic (PK) biomarkers and lymphocyte count, but not Treg percentage, suggesting that careful optimization of Cy and MMF dosing may have the potential to support immune recovery, although this requires further validation in larger studies.

**Conclusion:**

The relationship of pharmacokinetic biomarkers to immune and clinical outcomes warrants further investigation in larger studies but holds promise for personalizing dosing of GVHD prophylaxis to improve patient outcomes after alloHCT.

## 1 Introduction

Post-transplant cyclophosphamide (PTCy) is standard-of-care prophylaxis of graft-versus-host disease (GVHD) for allogeneic hematopoietic cell transplantation (alloHCT) using both HLA-matched and HLA-haploidentical donors. Despite the success of PTCy, emerging data highlighted concerns about the toxicity associated with PTCy, including organ toxicity (1, 2), delayed engraftment (2, 3), and increased risk of infection (2, 4–8). Active attempts are being made to optimize the PTCy regimen by de-escalation of the cyclophosphamide (Cy) dose. Thus far, reduction in PTCy dosing has shown promise clinically in maintaining GVHD prevention (2, 9–11) while leading to less toxicity, faster engraftment and immune reconstitution, and fewer infectious complications (2). These results suggested a potential therapeutic window for PTCy that warrants further study.

Cy has complex metabolism (12), and thus, pharmacokinetic (PK) assessments are essential to capture the interindividual variability in Cy and its metabolites associated with dosing. Cy is commonly combined with mycophenolate mofetil (MMF) as GVHD prophylaxis, usually with either a calcineurin inhibitor or sirolimus. Both Cy and MMF are prodrugs, and a large variation of over twofold in the areas under the curve (AUCs) of the metabolites of Cy and MMF has been reported for IV administration (13). However, Cy and MMF are prescribed at weight-based doses (with MMF also generally being capped at a fixed dose of 1,000 mg/dose in patients >66 kg in weight). This dose-selection standard does not consider patient-specific factors such as kidney function, liver function, and genetic polymorphisms that may cause large variability in drug exposure. It is possible that variation in exposure to both drugs and their metabolites may have clinical implications.

Immune reconstitution (IR) is an important process that is associated with HCT outcomes (14, 15). PTCy has been shown in preclinical models and also clinically to facilitate preferential regulatory T cell (Treg) recovery (16–22), which is a component of the mechanisms by which it prevents GVHD. Preclinically, the effects on preferential Treg recovery are dose-dependent (19, 20). However, it is not clear how variations in drug exposure change the IR pattern. Therefore, we assessed early immune reconstitution and associated drug recovery from a pilot study of patients treated with PTCy. Our data demonstrated the feasibility of pharmacokinetic and immune monitoring at early HCT days and revealed the potential correlation between PK exposure and lymphocyte recovery.

## 2 Material and methods

### 2.1 Study approval and patient selection

This study was approved by the City of Hope Institutional Review Board (IRB no. 18358) and conducted in compliance with the Declaration of Helsinki. Written informed consent was obtained for all study participants. Patients ≥18 years of age undergoing their first HLA-haploidentical alloHCT with PTCy as GVHD prophylaxis were accrued. GVHD prophylaxis consisted of PTCy, tacrolimus or sirolimus, and MMF. PTCy was administered at a dose of 50 mg·kg^−1^·day^−1^ on days +3 and +4 using ideal body weight (IBW) or adjusted body weight 25 (ABW25) when actual body weight was greater than 125% of IBW. MMF was administered three times daily as a 2-hour infusion from day +5 at a dose of 15 mg·kg^−1^·dose^−1^ capped at 1,000 mg using actual body weight (ABW) and switched to oral administration when tolerated. Tacrolimus or sirolimus was also started on day +5. Granulocyte colony-stimulating factor (G-CSF)-mobilized peripheral blood stem cells (PBSCs) were used as the graft source.

### 2.2 Pharmacokinetic sample processing

The pharmacokinetics of Cy were measured on days +3 and +4 with each Cy dose. The blood samples for Cy and metabolites were collected at 2 (end of infusion), 4, 6, 12, 20, and 24 hours from the start of Cy infusion on both days. The pharmacokinetics of MMF were monitored on day +7. Blood samples for MMF metabolites were collected at 0 (pre-dose), 2, 2.5, 3, 5, and 6 hours from the start of MMF infusion on day +7. The samples were processed as soon as possible, within 4 hours, by centrifugation at 1,500 *g* for 10 min to separate plasma. The aliquoted plasma samples were stored at −80°C until analysis via liquid chromatography–mass spectrometry (LC-MS).

### 2.3 Liquid chromatography–mass spectrometry

The LC-MS/MS system consisted of an ExionLC HPLC system interfaced to an AB SCIEX QTRAP^®^ 6500+ system (Sciex, Marlborough, MA). Stock solutions for standards and internal standards including Cy (2 mg/mL), carboxycyclophosphamide (cepm; 1 mg/mL), *N*-dechloroethyl cyclophosphamide (dccy; 1 mg/mL), 4-ketocyclophosphamide (ketocy; 1 mg/mL), Cy-D4 (1 mg/mL), cepm-D4 (1 mg/mL), and dccy-D4 (1 mg/mL) were prepared in MeOH and kept at −20°C before use. The separation was achieved using a Zorbax 1.8 μm SB-C18 2.1 × 50 mm column (Agilent Technologies, Santa Clara, CA). Mobile Phase A was 0.1% formic acid in H_2_O, and Mobile Phase B was 0.1% formic acid in MeOH. The following gradient program was used: 15% B (0–0.1 min), 15% to 25% B (0.1–1.0 min), 25% to 65% B (1.0–5.0 min), 65% to 80% B (5.0–6.0 min), 80% to 15% B (6.0–6.5 min), and 15% B (hold 6.5–7.5 min). The total run time was 7.5 min. The injector was maintained at 15°C. The injection volume was 3 µL. The column thermostat was set to 40.0°C, and the solvent flow was maintained at 0.3 mL/min. Multiple reaction monitoring (MRM) data were acquired under positive ion mode. Calibration mix was prepared in blank plasma. The dynamic range of the assays of Cy and its metabolites were as follows: Cy, 0.96 to 307 µM; cepm, 0.085 to 27 µM; dccy, 0.126 to 40.3 µM; and ketocy, 0.09 to 14.5 µM.

The total and unbound concentrations of mycophenolic acid (MPA) and mycophenolic acid glucuronide (MPAG) in plasma were quantified. Separation was achieved using a Zorbax 5 μm SB-C18 2.1 × 150 mm column (Agilent Technologies). Mobile Phase A was 0.1% formic acid in H_2_O, and Mobile Phase B was 0.1% formic acid in acetonitrile. The following gradient program was used: 30% B (0–0.2 min), 30% to 40% B (0.2–2.0 min), 40% to 50% B (2.0–2.5 min), 50% B (2.5–4.8 min), 50%–90% B (4.8–4.9 min), 90% B (4.9–6.4 min), 90%–30% B (6.4–6.5 min), and 30% B (hold 6.5–8.0 min). The total run time was 8 min. The injector was maintained at 15°C. The injection volume was 5 µL. The column thermostat was set to 37.0°C, and the solvent flow was maintained at 0.4 mL/min. Stock solution standards and internal standards, including MPA (1 mg/mL), MPAG (1 mg/mL), MPA-D3 (1 mg/mL), and MPAG-D3 (1 mg/mL), were prepared in MeOH and kept at −20°C before use. MRM data were acquired under positive and negative ion modes for MPA and MPAG, respectively. The calibration mix was prepared in blank plasma. The dynamic range of the assays of Cy and its metabolites were as follows: total MPA, 0.31 to 78 µM; total MPAG, 2 to 504 µM; unbound MPA, 0.006 to 2 μM; and unbound MPAG, 0.04 to 20 μM.

For total concentration, plasma samples were prepared by protein precipitation. Plasma samples or calibrators were mixed with an internal standard and 3 volumes of acetonitrile. Samples were vortexed and centrifuged to precipitate protein. The supernatant was diluted in water and loaded into the autosampler for injection. For unbound fractions of MPA and MPAG, an ultrafiltration method was adopted from previous publications (23, 24). Briefly, plasma samples were centrifuged at 2,000 *g* for 30 min at room temperature using an Amicon™ ultrafiltration device with a 30-kDa molecular cutoff (Millipore, Burlington, MA). The flowthroughs were mixed with internal standard, diluted with water, and loaded into the autosampler for injection. Plasma concentrations below the assay detection limit were treated as missing.

LC–MS peak integration and concentration calculation were performed in MultiQuant 3.0.3 (Sciex, Marlborough, MA). The interday precision was less than 10%. AUCs were calculated using the trapezoidal rule.

### 2.4 Immune cell monitoring by flow cytometry

T-cell subsets were monitored by flow cytometric analysis of blood samples collected on days +3 (prior to PTCy), +5 (prior to MMF and tacrolimus/sirolimus), +7, +14, and +21 after transplant. To obtain distributions of T, B, and natural killer (NK) cells in whole blood, blood samples were lysed with red blood cell lysis buffer (Gibco, Billings, MT) immediately after collection. The lysed blood samples were centrifuged, and total blood cells were collected and stained for cell surface markers. The antibody panel used was as follows: FITC anti-CD45 (clone HI30, BioLegend, San Diego, CA), PE anti-CD56 (clone B159, BD, Frankin Lakes, NJ), PerCP/Cy5.5 anti-CD16 (clone 3G8, BioLegend, San Diego, CA), PE/Cy7 anti-CD14 (clone 61D3, Invitrogen, Waltham, MA), APC anti-CD19 (clone HIB90, Biolegend, San Diego, CA), Alexa700 anti-CD3 (clone UCHT1, BD, Frankin Lakes, NJ), APC/Cy7 anti-CD4 (clone RPA-T4, Biolegend, San Diego, CA), and eFluor 450 anti-CD8 (clone RAP-T8, Invitrogen). Concentrations of each subset in blood were calculated in comparison with the clinical absolute lymphocyte count or white blood cell count that day.

To obtain data for T-cell subsets, whole blood was subjected to density centrifugation (Ficoll-Paque, Cytiva, Marlborough, MA) and centrifuged at 1,250 rpm for 30 min without break. The middle layer containing peripheral blood mononuclear cells (PBMCs) was collected, washed, treated with human FcR block (Human TruStain FcX) and stained with LIVE/DEAD Fixable Aqua Dead Cell Stain Kit (Thermo Fisher, Waltham, MA) and cell surface markers. The stained cells were fixed and permeabilized (eBioscience Foxp3 Staining Kit) and then stained with intracellular markers. The antibody panel used was as follows: AF700 anti-CD3 (clone UCHT1, BioLegend), BUV737 anti-CD4 (clone SK3, BD), PerCP/Cy5.5 anti-CD8 (clone SK1, BioLegend), BUV805 anti-CD14 (clone MSE2, BD), BV650 anti-CD15 (clone HIM1/HI98, BD), PE/Cy7 anti-CD25 (clone BC96, Invitrogen), APC/Cy7 anti-CD45RA (clone HI100, BioLegend), BV786 anti-CD69 (clone FN50, BD), PE/CF594 anti-CD95 (clone DX2, BD), BV605 anti-CD56 (clone 5.1H11, BioLegend), BV711 anti-CCR7 (clone G043H7, BioLegend)), APC anti-CD16 (clone 3G8, BD), PE anti-ICOS (clone C398.4A, BioLegend), BUV395 anti-HLA-DR (clone G46-6, BD), eFluor 450 anti-FoxP3 (clone PCH101, Invitrogen), and FITC anti-Ki-67 (clone B56, BD).

Flow cytometry data acquisition was carried out on a five-laser spectral flow cytometer (Cytek Aurora). The flow cytometry events were unmixed in SpectroFlo (Cytek, Fremont, CA). The gating of unmixed flow cytometry data was performed in FCS Express 7 (*De Novo* Software, Pasadena, CA). Additional analysis of T-cell subsets was conducted in RStudio (R version 4.3.0).

### 2.5 Statistics

All statistical analyses were performed using the R software (version 4.3.0). Cell count data were preprocessed by replacing zero values with the smallest value in the dataset that was not zero. Numerical variables were summarized as median and range. Group comparisons were conducted using the non-parametric Mann–Whitney U test. For correlation analysis, results from Spearman’s and Pearson’s correlations were reported. Lymphocyte counts were log-transformed before correlation analysis. Ties were resolved by adding a small amount of jitter to the dataset. Bootstrapping (1,000 iterations) was further performed to validate the association to increase the reliability of the analysis in the context of a small sample size. Statistically significant correlations were reported when p < 0.05 and also validated by bootstrapping.

Events from T-cell subsets were down-sampled and pooled after import into RStudio. Fluorescence intensity data were arcsin-transformed to distinguish positive from negative populations, with the cofactor manually selected by the researcher and verified using visualized plots in FCS Express to ensure accuracy. Dimensionality reduction was performed using selected markers to generate a Manifold Approximation and Projection (UMAP) embedding using the “umap” package. Positive populations for each surface or intracellular marker were identified by thresholding the transformed fluorescence intensity data and encoded into red, green, and blue channels. Colors on the UMAP were automatically generated based on the expression levels of the corresponding color-coded markers.

## 3 Results

### 3.1 Patient population and clinical outcomes

The patient characteristics are summarized in **Table 1**. Among the 11 patients, the median age was 33 years (range 20–72), including two women and nine men. Ten of the 11 patients received PTCy, tacrolimus, and MMF, and one patient received PTCy, sirolimus, and MMF as GVHD prophylaxis. Five patients developed acute GVHD (overall Grade I, n = 1; Grade II, n = 4) at a median of 59 (range 28–126) days. One patient had *de novo* mild chronic GVHD. One more patient initially presented with acute GVHD (aGVHD) and later developed severe chronic GVHD. One patient had poor graft function and had received sirolimus instead of tacrolimus. Overall, no relapse occurred among all patients during the follow-up period (**Table 2**), and a total of one patient (9.1%) died during the study period (follow-up locked as of day +58) (**Table 2**).

**Table 1:** Patient and transplant characteristics.

Patient specific variables		Stats
Number of patients		11
Recipient age, years (range)		32 (20–72)
Male sex		9 (82%)
Actual body weight (kg)		91.7 (55.7–117.4)
Ideal body weight (kg)		71.7 (54.7–79.9)
Disease	Acute myeloid leukemia	5 (45%)
	Acute lymphoblastic leukemia	4 (36%)
	Non-Hodgkin lymphoma	1 (9%)
	Myelodysplastic syndrome	1 (9%)
Conditioning intensity	Myeloablative	7 (64%)
	Reduced intensity	4 (36%)
Conditioning regimens	Fludarabine/TMLI	4 (36%)
	Fludarabine/FTBI	3 (27%)
	Melphalan/fludarabine/TBI	3 (27%)
	Fludarabine/cyclophosphamide/TBI	1 (9%)
GVHD prophylaxis	PTCy, tacrolimus, MMF	10 (91%)
	PTCy, sirolimus, MMF	1 (9%)
Donor age, years (range)		25 (19–34)
Donor–Recipient sex	Male to male	5 (45%)
	Female to male	4 (36%)
	Female to female	2 (18%)
Donor–recipient relationship	Siblings	6 (55%)
	Children	5 (45%)
ABO incompatibility	Major	5 (45%)
	Identical	4 (36%)
	Minor	1 (9%)
	Bidirectional	1 (9%)
Graft type	Peripheral blood stem cells	11 (100%)
Graft dose	CD34^+^ cells/kg (×10^6^ dose)	4.99 (4.36–5.51)
	CD3^+^ cells/kg (×10^8^ dose)	2.46 (1.31–4.6)

TMLI, total marrow and lymphoid irradiation; FTBI, fractionated total body irradiation; TBI, total body irradiation; PTCy, post-transplant cyclophosphamide (50 mg·kg^−1^·day^−1^ on days +3 and +4); MMF, mycophenolate mofetil.

**Table 2:** Post-transplant pharmacologic biomarkers and clinical outcomes.

Patient	Cyclophosphamide AUC_0–48hr_ (µM × hr)				Total AUC_0–6hr_ (µM × hr)		Unbound AUC_0–6hr_ (μM × hr)		GVHD outcome (days)	Lymphocyte day +30 (counts/μL)	Alive	Relapse	Follow-up (months)
	Cy	Cepm	Dccy	Ketocy	MPA	MPAG	MPA	MPAG					
1	3,414	298	252	167	30.6	156	0.31	22.2		790	Yes	No	24
2	4,287	178	282	93	30.7	198	0.31	31.3	aGVHD: day +59; cGVHD: day +164	1,368	Yes	No	23
3	2,435	342	141	153	61.4	387	0.81	61.1	aGVHD: day +62	524	Yes	No	21
4	3,373	294	181	100	68.8	384	0.63	56.1		658	Yes	No	21
5	3,543	307	272	132	37.8	300	0.40	46.3	aGVHD: day +31	290	Yes	No	20
6	3,716	533	332	169	76.4	826	1.06	122.9	aGVHD: day +28	0	Yes	No	19
7	2,174	499	323	271	79.4	644	0.86	100.3	cGVHD: day +132	79.9	Yes	No	17
8	3,831	210	209	211	53.9	285	0.56	38.9		240	Yes	No	16
9	2,882	228	68	131	57.4	254	0.81	34.3	aGVHD: day +126	208	Yes	No	13
10^a^	5,657	3,404	853	207	120.0	1,817	1.85	325.5		10.3	No	No	2
11	2,581	355	174	135	33.2	148	0.26	19.1		222	Yes	No	10
Median	3,414	307	252	153	57.4	300	0.63	46.3		Fast: 407 (208–1,368)			
(range)	(2,174–5,657)	(178–3404)	(68–853)	(93–271)	(30.6–120)	(148–1817)	(0.26–1.85)	(19.1–325)		Slow: 10.3 (0–79.9)			
Fold range^b^	2.6	19.1	12.5	2.9	3.9	12.3	7.1	17.1					
	2.0	3.0	4.9	2.9	2.6	5.6	4.1	6.4					

AUC, area under the curve; GVHD, graft-versus-host disease; aGVHD, acute graft-versus-host disease; cGVHD, chronic graft-versus-host disease; Cy, cyclophosphamide; Cepm, carboxycyclophosphamide; Dccy, *N*-dechloroethyl cyclophosphamide; Ketocy, 4-ketocyclophosphamide; MPA, mycophenolic acid; MPAG, mycophenolic acid glucuronide.

a Patient had poor graft function; patient had not had GVHD at the time of death.

b Fold range values were calculated from all 11 patients (first row) and 10 patients excluding ID#10 (second row).

### 3.2 Lymphocyte and T-cell recovery after HCT

**Figure 1:**
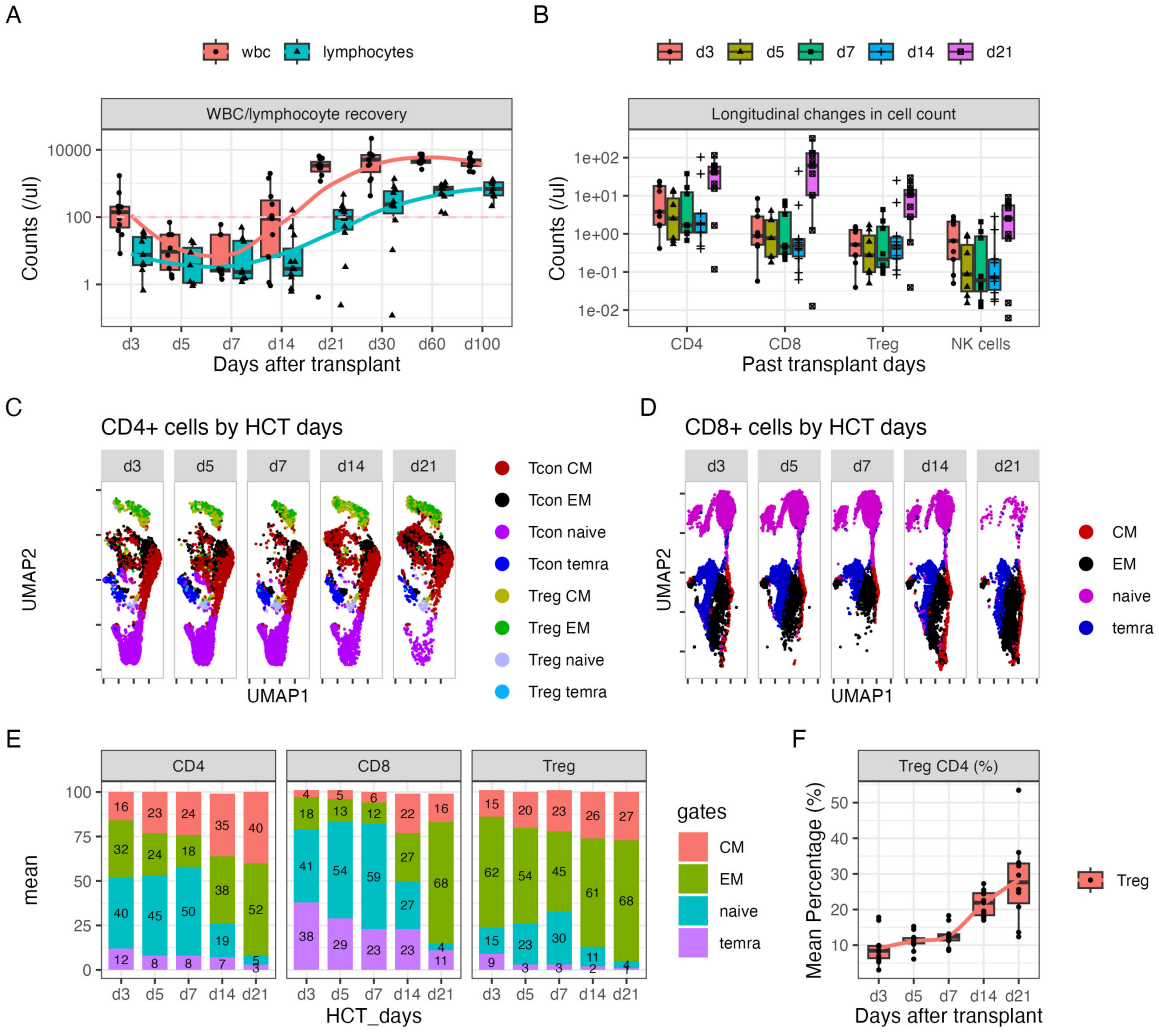
Dynamic changes in CD4^+^ and CD8^+^ subsets over the first 21 days after HLA-haploidentical transplant. **(A, B)** Blood concentrations (cells/µL) of **(A)** white blood cells and lymphocytes and **(B)** CD4^+^, CD8^+^, CD4^+^ regulatory T cells (Tregs; CD25^+^Foxp3^+^ within CD4^+^ T cells), and natural killer (NK) cells serially over the first 100 days after transplant. **(C, D)** UMAP plots visualizing of naïve (lavender), central memory (red), effector memory (black), and CD45^+^ terminally differentiated T effector memory (TEMRA) (blue) cells in **(C)** CD4^+^ and **(D)** CD8^+^ T-cell subsets; Tregs predominantly clustered in the top region, indicated by green [effector memory (EM)], yellow [central memory (CM)], and light blue (TEMRA) colors. Additionally, a subset of naïve Tregs was observed in the gray cluster adjacent to the naïve cell population in panel **(C)**. **(E)** The average percentage of naïve, CM, EM, and TEMRA cells in CD4^+^, CD8^+^, and Treg subsets. The numbers on the bar represent the percentage expressed as mean. **(F)** The average percentage of Tregs in the CD4^+^ population over time.

The longitudinal recovery of white blood cells (WBCs), lymphocytes, CD4^+^ T cells, CD8^+^ T cells, Tregs, and NK cells is shown in **Figures 1A, B**. The concentrations of blood cells decreased until day +7 and recovered beginning at day +14. Eight patients (72.8%) had lymphocyte counts at a median (range) of 407 (208–1,368) cells per microliter at day 30, while the other three patients (27.2%) had lymphocyte counts at 10 (0–80) cells per microliter at day 30. The recovery of CD4^+^ and CD8^+^ T cells over the first 3 weeks followed a similar trend as lymphocytes, with faster recovery of Tregs compared with other subsets at day +14 (**Figure 1B**).

### 3.3 Dynamic change in T-cell subsets after HCT

Seventeen-parameter flow cytometry of T-cell subsets was performed on days +3, +5, +7, +14, and +21 after transplant. On average, 40% of CD4^+^ cells, 41% CD8^+^ cells, and 15% Tregs showed a naïve phenotype on day +3 (**Figures 1C–E**). The percentage of naïve cells slightly increased through day +7 and precipitously decreased by day +14 for all three populations (**Figure 1E**). The majority of cells showed a memory phenotype. Moreover, the percentage of Tregs steadily increased through day +21, with the average percentage of Tregs on days +14 and +21 being 22% and 28%, respectively (**Figure 1F**).

**Figure 2:**
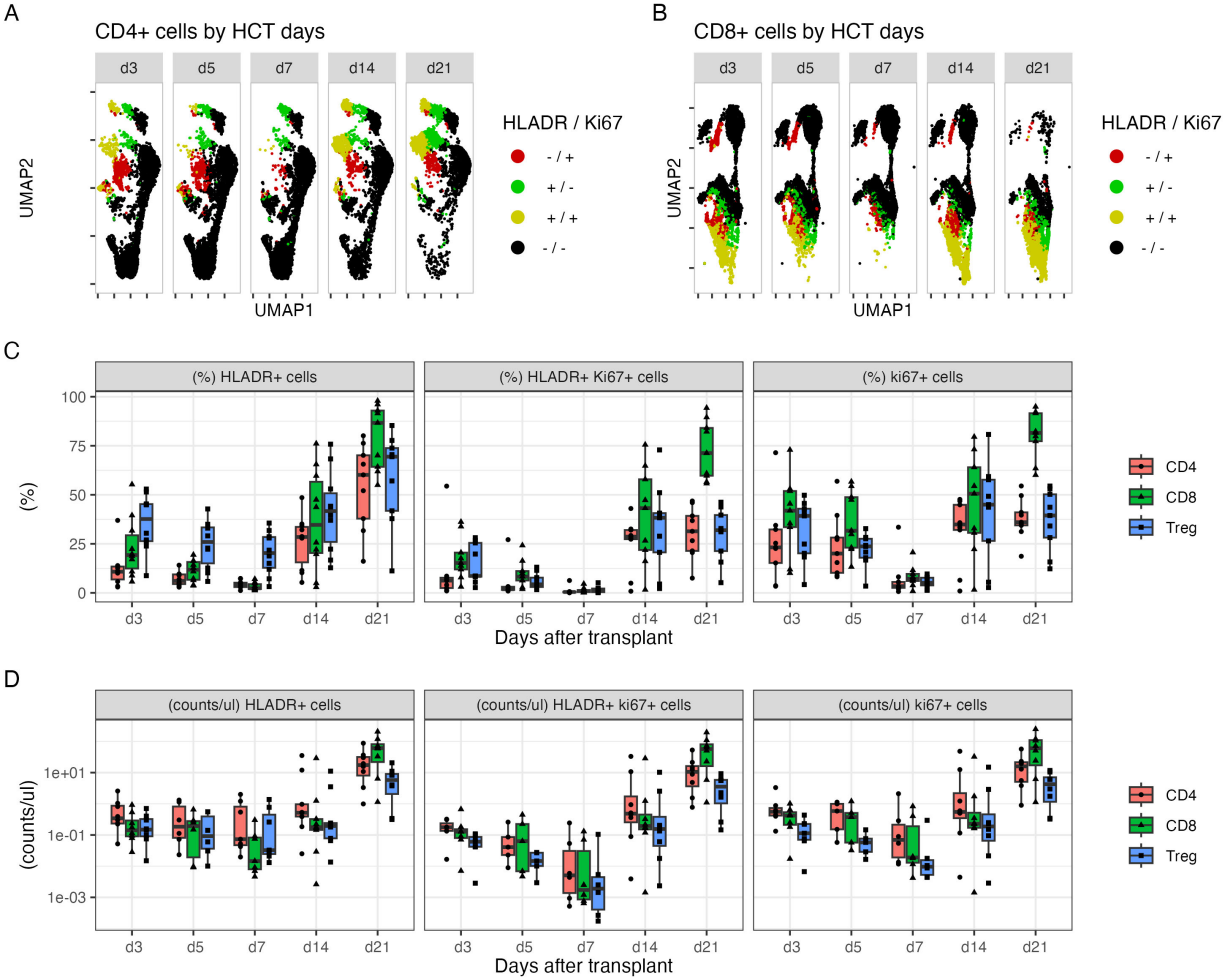
The dynamic changes in subpopulations of CD4^+^ and CD8^+^ cells within 21 days after HLA-haploidentical transplant. **(A, B)** UMAP plots visualizing of HLA-DR^+^ (green), Ki-67^+^ (red), and double-positive cells (yellow) in **(A)** CD4^+^ and **(B)** CD8^+^ populations. **(C)** The average percentage (%) and **(D)** blood concentrations (cells/µL) of HLA-DR^+^, Ki-67^+^, and double-positive CD4^+^ T cells (red), CD8^+^ T cells (green), and Tregs (blue).

The expression of Ki-67, HLA-DR, and CD69 on T-cell subsets was simultaneously monitored. There was an acute reduction in the numbers of Ki-67^+^ and HLA-DR^+^ cells, particularly the double-positive population on day +5 and particularly day +7, compared to that on day +3, in the CD4^+^ (**Figures 2A, C**), CD8^+^ T-cell (**Figures 2B, C**), and Treg populations (**Figures 2A, C**). All three populations increased thereafter. By day +21, CD8 T cells had the highest percentage of all three cell types (**Figures 2C, D**). However, CD69 expression on the three cell types did not change between days +3 and +7 (**Supplementary Figure S1**).

### 3.4 Variation in drug exposure and its correlation with T-cell recovery

**Figure 3:**
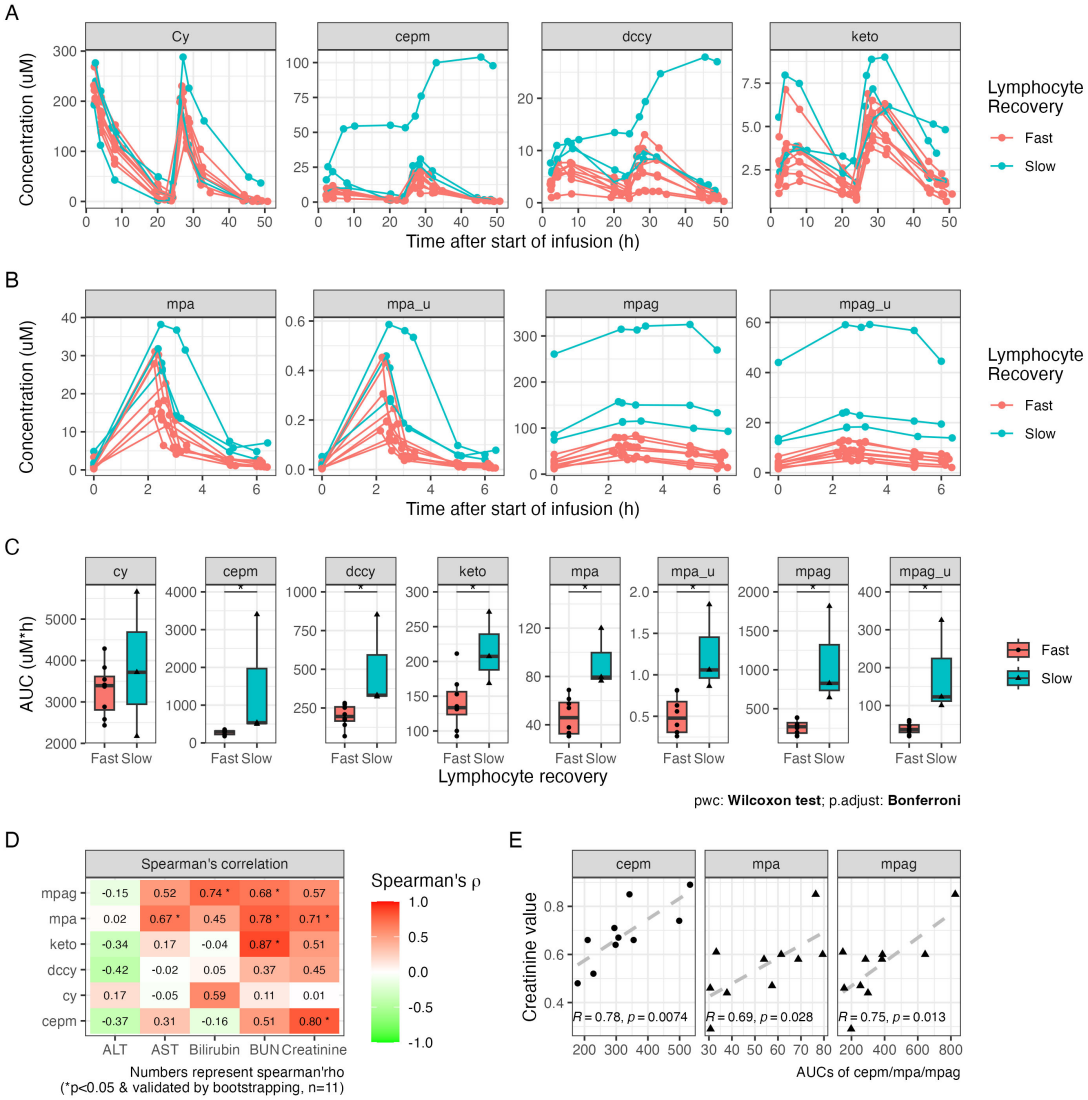
Plasma drug exposure after HLA-haploidentical transplant. **(A)** The plasma concentrations of cyclophosphamide (Cy), carboxycyclophosphamide (cepm), *N*-dechloroethyl cyclophosphamide (dccy), and 4-ketocyclophosphamide (keto). **(B)** The plasma concentrations of MMF metabolites include total and unbound mycophenolic acid (MPA; total: mpa, unbound: mpa_u) and mycophenolic acid glucuronide (MPAG; total: mpag, unbound: mpag_u). **(C)** The AUCs of Cy and MMF metabolites in subgroups with fast and slow lymphocyte recovery, defined by lymphocyte count on day +30. **(D)** Tile image of color-coded correlation coefficient calculated between the AUCs on the y-axis and ALT, AST, bilirubin, BUN, and creatinine on the x-axis. The fill color and number on each tile represent correlation coefficient. Statistical significance (*) was reported when p < 0.05 and was also validated by bootstrapping. Sample size is indicated as n. **(E)** Scatter plots of the AUCs of cepm, total MPA, and total MPAG on the y-axis against creatinine values on the x-axis after excluding outliers. Pearson’s correlation coefficient (R) and p-value (p) are shown at the bottom of the plot. MMF, mycophenolate mofetil; AUC, area under the curve; ALT, alanine aminotransferase; AST, aspartate aminotransferase; BUN, blood urea nitrogen.

The plasma concentration profile of Cy and its metabolites, as well as MPA and MPAG, varied widely among individuals (**Figures 3A, B**). One outlier in the pharmacokinetic profile was identified, who was the patient with poor graft function and who showed distinct profiles of Cy, cepm, dccy, MPA, and MPAG AUCs, all much higher compared to those of the others. The AUCs from each study subject and the summary of AUCs and fold difference are shown in **Table 2**. The interindividual variability, measured by fold difference in AUCs excluding the above-mentioned outlier, was 2-fold for Cy, 3-fold for cepm, 4.9-fold for dccy, 2.9-fold for ketocy, 2.6-fold for total MPA, and 5.6-fold for total MPAG (**Table 2**). When the drug exposures were evaluated relative to the pace of lymphocyte recovery, all three patients who had low lymphocyte counts on day 30 had higher exposure to all metabolites, including cepm, dccy, ketocy, MPA, and MPAG (**Figure 3C**). When the drug exposures were evaluated relative to GVHD outcome, no distinct trends were observed in the AUCs of Cy, cepm, MPA, or MPAG between patients who later did or did not develop aGVHD (**Supplementary Figure S2**), although these analyses were limited by the small numbers of patients. To assess factors that could be contributing to the variability in drug exposure, a correlation analysis was performed between AUCs and biological parameters of renal or hepatic function, including blood urea nitrogen (BUN), creatinine, bilirubin, alanine aminotransferase (ALT), and aspartate aminotransferase (AST). The correlation analysis was conducted between AUCs and these blood markers on day +4 for Cy metabolites and day +7 for MMF metabolites when the blood was sampled for the concentrations of Cy and MMF, respectively. Creatinine showed positive correlations with the AUCs of cepm (Spearman’s rho = 0.80, 95% bootstrap CI: 0.29–1, n = 11) and MPA (Spearman’s rho = 0.71, 95% bootstrap CI: 0.17–0.97, n = 11). BUN showed a positive correlation with AUCs of MPAG (Spearman’s rho = 0.68, 95% bootstrap CI: 0.06–1.0, n = 11), MPA (Spearman’s rho = 0.78, 95% bootstrap CI: 0.19–0.99, n = 11), and ketocy (Spearman’s rho = 0.87, 95% bootstrap CI: 0.40–1, n = 11). Bilirubin and AST were positively correlated with AUCs of MPA and MPAG, respectively (**Figure 3D**). Pearson’s correlation also showed a positive association between the above-mentioned PK biomarkers and BUN and creatinine levels (**Figure 3E**, **Supplementary Figure S3**), suggesting a moderate association between renal function and PK biomarkers.

### 3.5 Pharmacokinetic biomarkers associated with immune reconstitution

**Figure 4:**
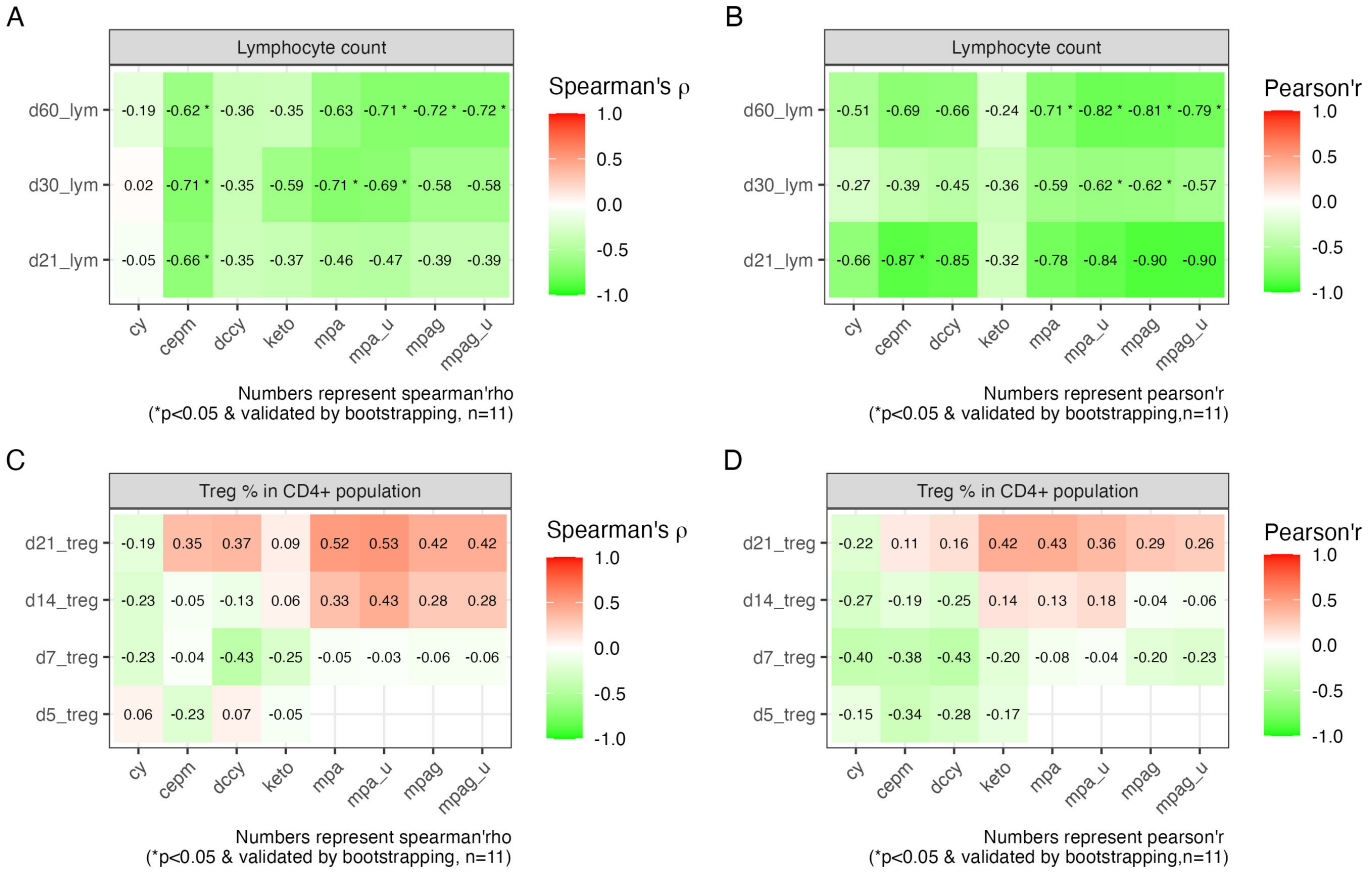
Correlation between PK biomarkers and lymphocyte recovery and Treg percentage. **(A)** Tile image of color-coded correlation coefficient calculated between the AUCs on the x-axis and lymphocyte count on different HCT days indicated on the y-axis using Spearman’s correlation and **(B)** Pearson’s correlation. **(C)** Tile image showing correlation between AUCs on the x-axis and Treg percentage on different HCT days indicated on the y-axis using Spearman’s correlation and **(D)** Pearson’s correlation. The fill color and number on each tile represent correlation coefficient. Statistical significance (*) was reported when p < 0.05 and further validated by bootstrapping. Sample size is indicated as n. PK, pharmacokinetic; AUC, area under the curve; HCT, hematopoietic cell transplantation.

To further investigate the association between pharmacokinetic biomarkers and immune cell recovery, we conducted correlation analysis using Pearson’s and Spearman’s methods. The result showed that the AUCs of cepm were negatively correlated with lymphocyte count on day +21 (Spearman’s rho = −0.66, 95% bootstrap CI: −0.99 to −0.04, n = 11), day +30 (Spearman’s rho = −0.71, 95% bootstrap CI: −0.97 to −0.1, n = 11), and day +60 (Spearman’s rho = −0.62, 95% bootstrap CI: −0.92 to −0.04, n = 11). The findings from Pearson’s correlation were consistent with those from Spearman’s correlation on day +21 (**Figures 4A, B**). Lymphocyte count was also negatively associated with unbound MPA on days +30 and +60, and with total and unbound MPAG on day +60, as confirmed by both Spearman’s and Pearson’s correlation analysis (**Figures 4A, B**). However, bootstrap resampling (1,000 iterations) yielded 95% confidence intervals that spanned a wide range in the correlation direction, highlighting some uncertainty in the strength of the association. The percentage of Tregs is not significantly correlated with any of the PK biomarkers (**Figures 4C, D**).

## 4 Discussion

The current study aimed to estimate the interindividual variability in drug exposures and explore the relationship between drug exposure and clinical or immune outcomes in HLA-haploidentical alloHCT patients. We monitored dynamic changes in T-cell subsets, especially Tregs, in the first 3 weeks after alloHCT in relation to the administration of PTCy, MMF, and either tacrolimus or sirolimus. Consistent with previous reports (13, 25), we observed moderate inter-patient variability in both Cy and MMF metabolites, but with an outlier exhibiting markedly higher drug exposure. PTCy treatment coincides with a temporary decrease in proliferating T cells and a steady increase in Treg frequency. Additionally, a significant negative correlation was observed between AUCs and lymphocyte counts, but not the percentage of CD4^+^ T cells that were Tregs. While these results suggest the potential predictive value of PK biomarkers for immune recovery, this observational study had a small sample size (n = 11) and did not include an interventional cohort for comparison. Validation in larger, interventional cohorts will be essential to confirm these findings.

Tregs are important mediators in GVHD prevention. Preferential reconstitution of Tregs at day +21 has been reported in mice treated with PTCy and is associated with the effectiveness of GVHD prevention (21). However, evidence regarding Treg dynamics in humans early after transplantation remains scarce. In this study, we monitored the dynamic changes in T-cell subsets early in relation to the initiation of PTCy. The result showed on day +3 before PTCy that the majority of CD4^+^ and CD8^+^ T cells exhibited a naïve phenotype, whereas Tregs predominantly displayed a memory phenotype. Proliferation was mainly observed in memory and terminally differentiated T effector memory (TEMRA) cells, but not naïve T-cell phenotypes. PTCy treatment coincided with a transient reduction of proliferating T cells. However, the frequency of Tregs and all naïve T cells slightly increased on days 5 and 7, supporting previous findings that both Tregs and naïve CD8^+^ T cells may be more resilient to Cy treatment, even if there are discrepant *in vitro vs*. *in vivo* data on naïve CD4^+^ T cells (16, 26, 27). Prior study found a higher frequency of Tregs in HLA-haploidentical alloHCT patients receiving PTCy and sirolimus compared to healthy controls (18). Consistently, we found that Treg frequency continued to rise throughout the first 21 days post-transplant. This finding supports prior observations of preferential Treg recovery following PTCy (16, 19–21). Moreover, variability in PK biomarkers was not associated with Treg frequency. The AUCs also did not differ across GVHD strata (**Supplementary Figure S2**), consistent with prior studies (2, 28). These results suggest that decreasing the current dose of Cy may not substantially compromise the preferential recovery of Tregs and the associated GVHD prevention, which is consistent with positive outcomes reported from clinical trials using lower doses of Cy (2, 9–11). However, further validation in larger cohorts is needed to confirm these observations.

Aside from GVHD prevention, adequate and timely immune recovery is critical for post-transplant outcomes. Several retrospective studies have reported that inadequate lymphocyte recovery (less than 200–300/μL) at day 30 or later is associated with fewer optimal outcomes (29–32). In the current study, three of the 11 patients had low lymphocyte count on day 30 (<80/μL) compared to the rest (>200/μL). Additionally, these patients also experienced higher exposure to Cy and MMF metabolites. Indeed, PK biomarkers, including cepm, unbound MPA, and MPAG, were negatively associated with lymphocyte count. Although larger interventional studies are needed to determine the nature of this association, the known pharmacological actions of the active metabolites of Cy and MMF suggest that they would have a potential negative influence on lymphocyte recovery. MPA, the active form of MMF, inhibits T-cell proliferation by selectively and reversibly inhibiting inosine monophosphate dehydrogenase (IMPDH) (33), and high doses of MMF may exert an undesired effect on engraftment (34). Similarly, reactive metabolites of Cy, including 4-hydroxycyclophosphamide, can induce chromosomal damage, and higher than necessary exposure levels may cause delayed immune recovery. Cepm is a stable metabolite formed from the 4-hydroxycyclophosphamide pathway. Cepm is not toxic itself but may serve as a biomarker of intracellular exposures to the cytotoxic metabolites (25, 35). Similar to cepm, MPAG is a metabolite of MPA and is inactive toward IMPDH and, therefore, is not cytotoxic (36). Consistently, recent results showed an inverse relationship between 4-hydroxycyclophosphamide exposure and T-cell recovery at days +14, +21, and +28 in patients receiving HLA-haploidentical bone marrow alloHCT (2). It is worth noting that among all patients, the three patients who had slow lymphocyte recovery had the highest AUCs of both MPA and MPAG. Intriguingly, these same patients also had the highest exposure to cepm and dccy. It is possible that other underlying factors, such as disease state or confounding variables, may contribute to both higher drug exposure and slower lymphocyte recovery. In this case, MPA and MPAG exposure may be modified similarly to cepm and thus serve as a biomarker of exposure to the other drug. However, this hypothesis requires validation in larger populations.

The goal of therapeutic monitoring is to identify the relationship between drug exposure and clinical outcomes, thus justifying personalized dose selection. Given the short duration of PTCy treatment at just two doses over 2 days, and the fact that Cy metabolism is subject to impact from autoinduction of cytochrome P450 within this time frame, dose adjustment based on real-time monitoring of AUCs may be challenging. Biomarker-guided dosing may offer advantages by forecasting drug exposure and enabling risk stratification during dose selection. Previously, studies have initiated the effort in exploring the feasibility of lipidomic and metabolomic markers of Cy and its metabolites (37, 38). However, no biomarker has been validated thus far, and future study is needed to explore the feasibility of this approach. Moreover, clinical practice is still grounded in weight-based dosing, and who would benefit the most from a lower dose is still open to discussion. Predicative factors or algorithms that can classify patients into strata corresponding to drug exposure and subsequent outcomes will be helpful to establish a more informed dose-selection criterion. Although Cy and MPA are mainly cleared through the hepatic pathway and are generally considered safe in patients with renal impairment (39, 40). However, renal function has been reported to impact the AUCs of Cy and its metabolites (41–43), as well as MPA and MPAG (44). Further investigation is needed to elucidate the nature of the association between renal function indicators (such as creatinine) and drug exposures, as well as to identify other factors contributing to the pharmacokinetic variability in both Cy and MMF, to explore their potential for biomarker-based dosing.

## Data Availability

The original contributions presented in the study are included in the article/**Supplementary Material**. Further inquiries can be directed to the corresponding author.
