# Supplementary figures and images for: Association of pharmacokinetic biomarkers with early immune recovery following HLA-haploidentical hematopoietic cell transplantation
Source: Front Immunol. 2025 Dec 5;16:1694754. doi: 10.3389/fimmu.2025.1694754 (PMC12714604; doi:10.3389/fimmu.2025.1694754)

## Slide 1
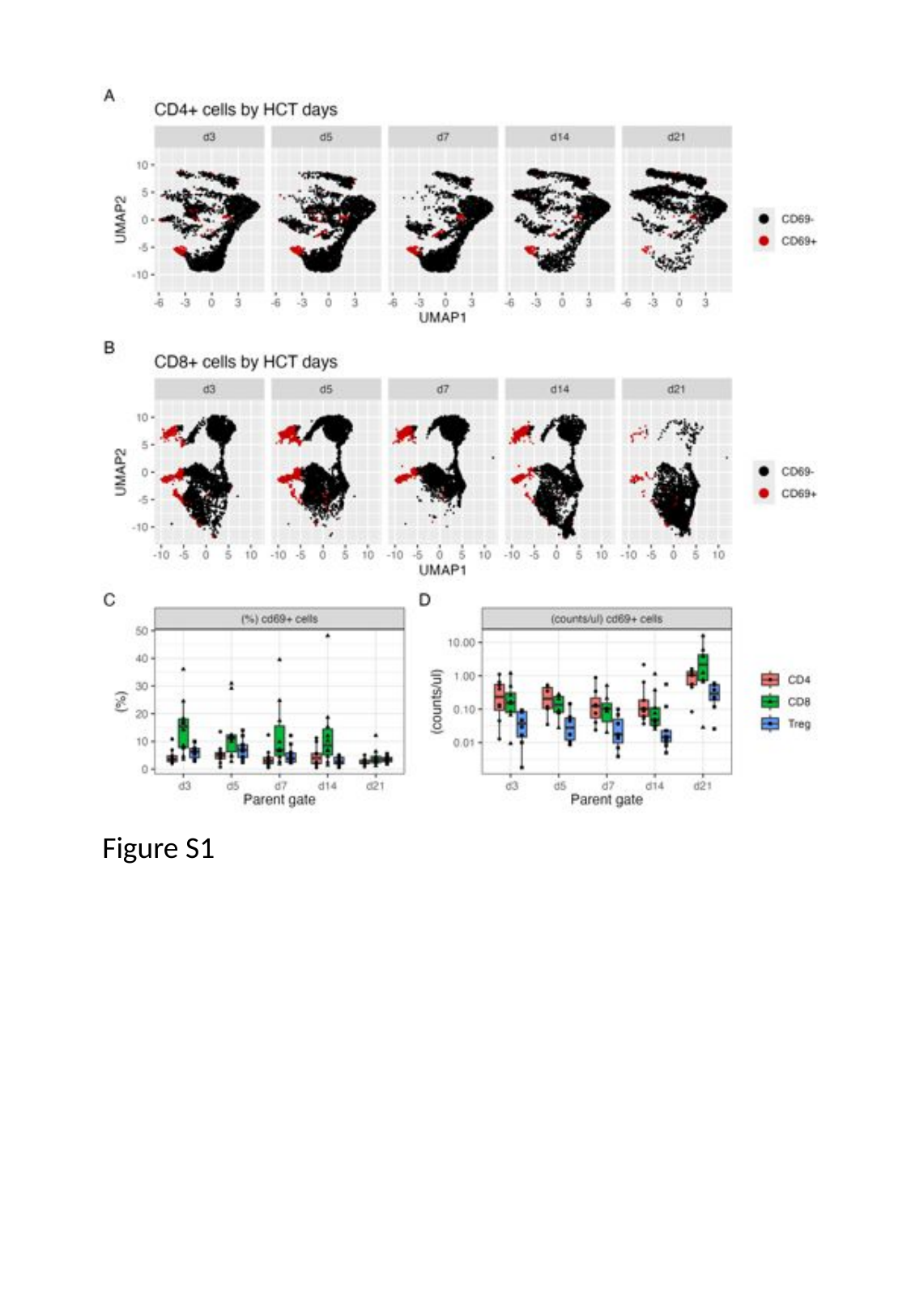

Figure S1

## Slide 2
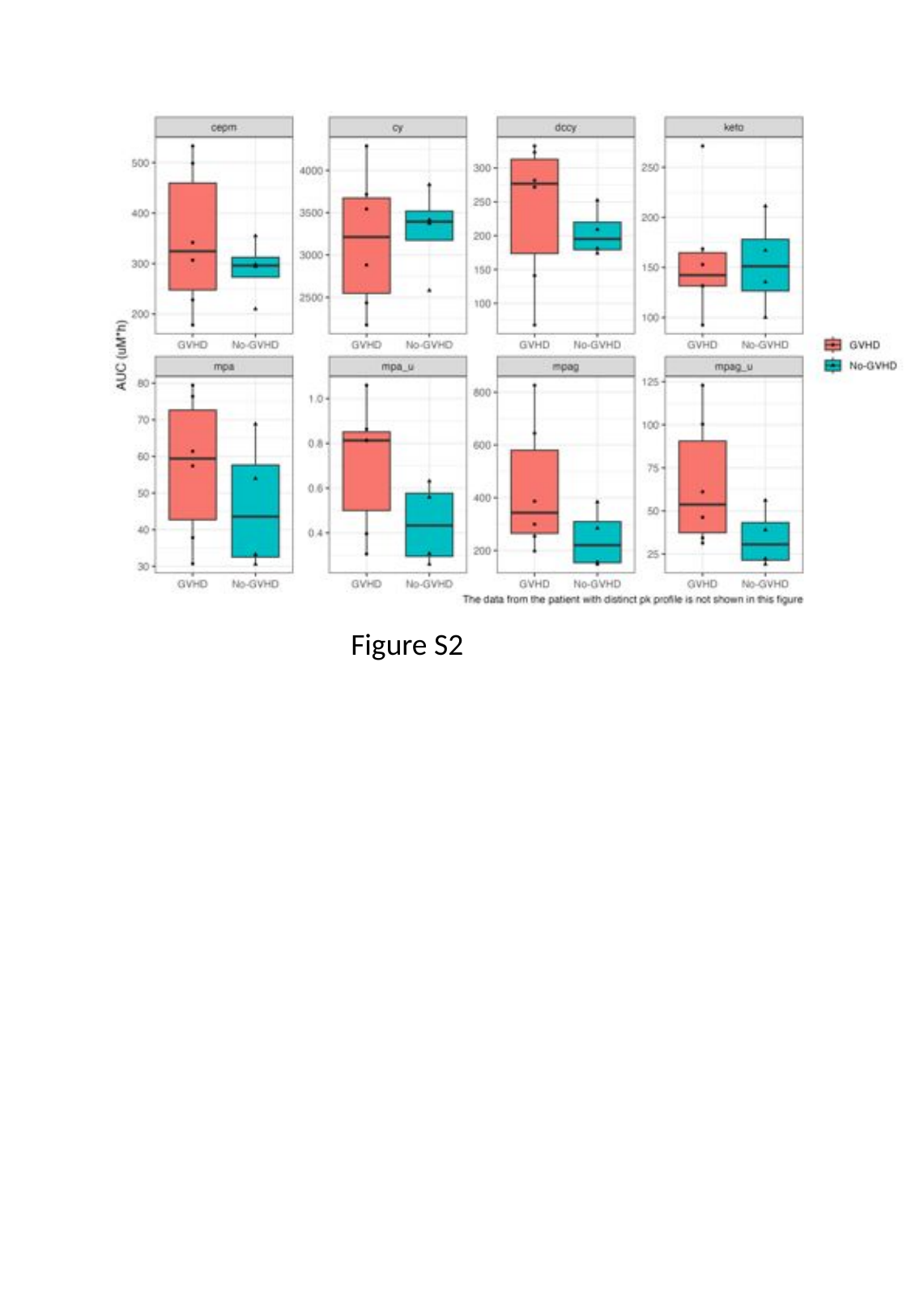

Figure S2

## Slide 3
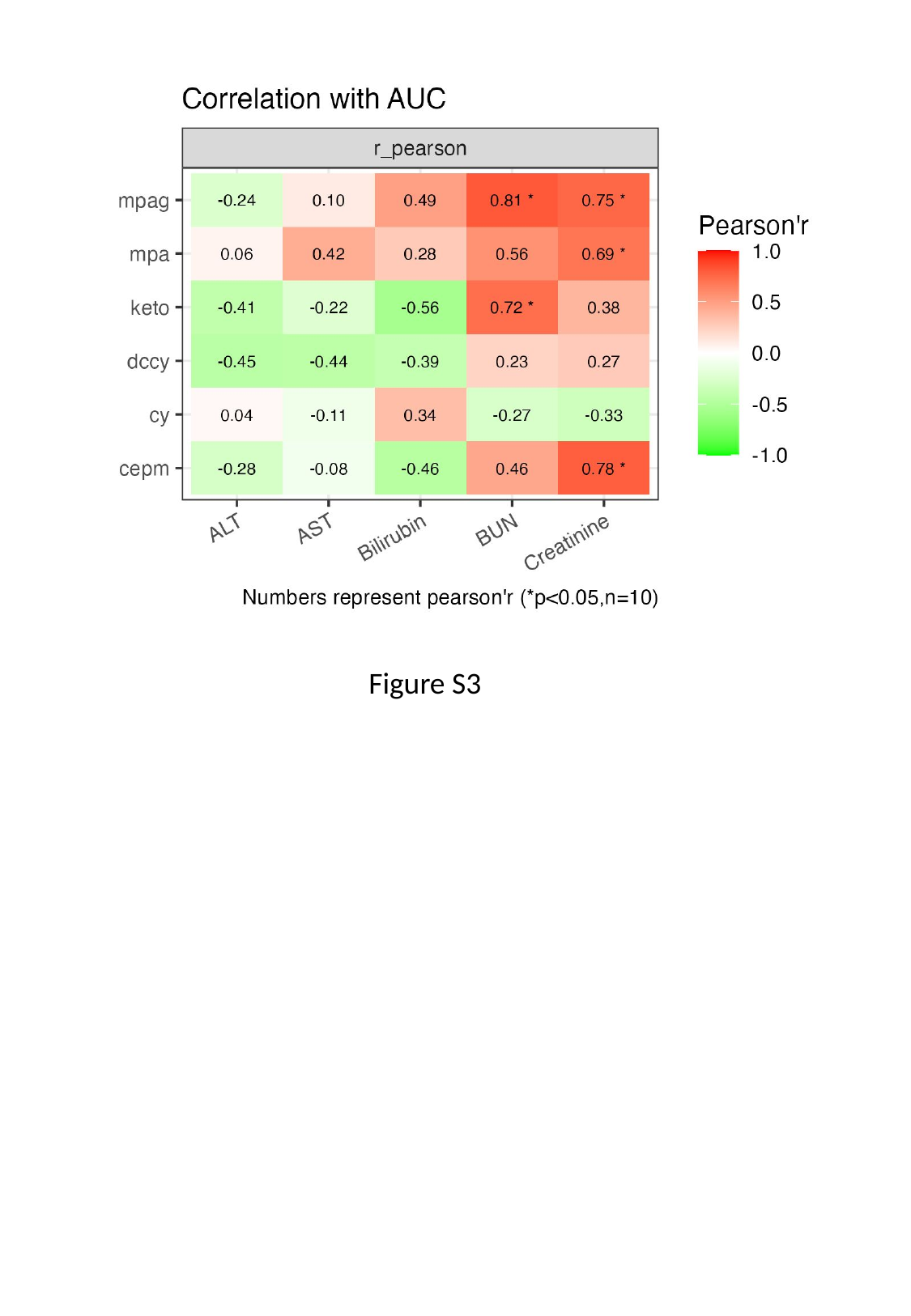

Figure S3

Supplement: Supplementary file 1 [file Presentation1.pptx]
